# Preliminary evaluation of a candidate international reference for Epstein–Barr virus capsid antigen immunoglobulin A in China

**DOI:** 10.1186/s13027-020-00294-8

**Published:** 2020-04-29

**Authors:** Hao Chen, Qiaohua Zhong, Xiaobin Wu, Yanling Ding, Qi Chen, Ning Xue, Yiwei Xu, Shulin Chen

**Affiliations:** 1 State Key Laboratory of Oncology in South China, Collaborative Innovation Center for Cancer Medicine, Guangdong Key Laboratory of Nasopharyngeal Carcinoma Diagnosis and Therapy, Sun Yat-sen University Cancer Center, Guangzhou, 510060 People’s Republic of China; 2 Department of Clinical Laboratory ,People’s Hospital of Jieyang, Jieyang Hospital Affiliated to SunYat-sen University, Tianfu Road 107, Rongcheng District, Jieyang City, 522000 Guangdong Province People’s Republic of China; 3 Department of Laboratory Science, The Second Affiliated Hospital of Guangzhou University of Chinese Medicine, Guangdong, People’s Republic of China; 4 Department of Clinical Laboratory, Liuzhou Maternity and Child Health Care Hospital, Yingshan Road 50th, Chengzhong District, Liuzhou, 545001 People’s Republic of China; 5 Department of Clinical Laboratory, Affiliated Tumor Hospital of Zhengzhou University, Henan Tumor Hospital, Zhengzhou, 450100 People’s Republic of China; 6 Department of clinical laboratory, The cancer hospital of Shantou University Medical college, The Key laboratory of Molecular Biology for high cancer incidence coastal Chaoshan area, Shantou University Medical college, number 22, Xinling road, Shantou, Guangdong 515041 People’s Republic of China

**Keywords:** Epstein–Barr virus, Nasopharyngeal carcinoma, Virus capsid antigen, Reference, Enzyme-linked immunosorbent assay (ELISA)

## Abstract

**Background:**

The detection of the Epstein–Barr capsid antigen (VCA) immunoglobulin A (IgA) is widely used in the diagnosis of nasopharyngeal carcinoma (NPC), but a reference standard for evaluating the presence of VCA-IgA is not yet available. Therefore, a reference standard is urgently needed for a uniform and quantitative detection of VCA-IgA.

**Methods:**

A mixed reference serum from three NPC patients diluted with healthy subject serum was made as a potential first international standard for VCA-IgA. VCA-IgA was detected in twenty NPC patients by four ELISA kits and two chemiluminescent immunoassays kits using the reference as a calibration curve. The performance of these six kits was evaluated, and the quantitative results were compared.

**Results:**

Our results showed a good linearity of the reference in different kits. Without reference, the difference of the total coefficient of variation (from 3.98 to 43.11%) and Within-run coefficient of variation (from 2.47 to 19.66%) was large in the 6 kits. The positive and negative coincidence rate between the 6 kits and indirect immunofluorescence for NPC diagnosis was 75% overall agreement, but a difference among the six kits was found, ranging from 55 to 90%. The concentration of VCA-IgA in the 20 NPC samples led in the division into three categories such as negative, low, or medium/high positive, but these concentrations were significantly different within these three categories depending on the kit used of the 6 considered. However,a good correlation (R^2^ = 0.986) was observed between Antu and Beier ELISA kits.

**Conclusions:**

The reference serum mightbe used as a reference standard for a better comparison of the results from different kits/laboratories. However, the quantitative results of some kits are still inconsistent due to the diversity of VCA antigens.

## Introduction

Nasopharyngeal carcinoma (NPC) is highly endemic in South China. The ASIRW (age-standardized incidence rates of world) in South China (9.69/100,000) was 3.4 times higher than that in Southwest China (2.85/100,000), which is the second in terms of incidence [1, 2]. Epstein–Barr virus (EBV) infection is one of the most important factors causing NPC onset [3]. EBV associated antibodies, such as virus capsid antigen (VCA-IgA) and Epstein-Barr virus nuclear antigen 1 (EBNA1-IgA), are used in the screening and diagnosis of NPC [4] and the former being one of the most widely used [5–8]. VCA-IgA antibody is usually measured by indirect immunofluorescence (IFA) [9] or enzyme-linked immunosorbent assay (ELISA) [10] with IFA considered as the “gold standard”. Thanks to the presence of enzyme-labeled antibodies instead of fluorescent antibodies, the immunoenzymatic assay (IEA) does not need a fluorescence microscope to interpret the results, which are semi-quantitatively reported by the titers and are widely used in southern China [2, 3].

The VCA-IgA titer is closely related to the longterm curative effect and prognosis of NPC [11, 12]. Low titer has a better therapeutic effect on NPC and prognosis, while ascending EBV antibody titers are strongly associated with an increased risk of NPC [13, 14]. Although the titer detected by traditional IEA or IFA has considerable prognostic value, these two approaches are not suitable for mass NPC screening because of the need for manual interpretation. ELISA-based assays have detection sensitivity and specificity similar to IFA [4, 15] but they have the advantage of simpler standardization, they are more tolerant to interference, and less time-consuming when a large number of specimens need to be analyzed. The chemiluminescent immunoassay (CLIA) has advantages similar to ELISA, it was developed in China, but no literature is yet available.

Thus, ELISA and CLIA are convenient and automated, but they use different peptide segments of VCA for their performance and standardization, thus, this difference is still a matter of debate and concern. Inaddition, while new methodologies and new platforms (automated vs.manual, ELISA vs. CLIA) for the detection of VCA-IgA are available, a proper comparisons among different assays has not yet been carried out. An important topic of discussion concerns the potential use of nature reference preparations to calibrate these assays for quantitative testing. In addition, aproper cross-validation and evaluation of the available different methods and kit brands are lacking.

So far, a reference standard for VCA-IgA has not yet been determined by the World Health Organization (WHO) International Laboratory and Collaborating Centers for Biological Standards. Most commercial ELISA kits generate only negative or positive results [16]. Although our previous study confirmed that the rOD value of ELISA shows a good correlation with the titer from IEA [2], at present, VCA-IgA detected by ELISA can not be used in the prognosis of NPC in place of that detected by IFA. Since VCA-IgA ELISA is widely used in clinical laboratories, a calibrator is urgently needed. Recently, a potential reference sample was proposed by Zhong He (Guangzhou, China) and Sun Yat-sen University Cancer Center (SYSUCC, Guangzhou, China) as a request of the Guangdong Provincial Anticancer Association (http://www.gdaca.org.cn/), Southern China Tumor Markers Standardization Alliance (Guangzhou, Guangdong, P.R.China). This sample was obtained from the blood serum of three NPC patients and mixed with the blood serum of healthy subjects for dilution purposes. This sample might become the first international VCA-IgA reference only if it fulfills specific criteria essential for such use. Therefore, this study was performed in six clinical laboratories to evaluate the quantitative detection of VCA-IgA by different immunoassays to verify whether the results could be improved by the use of this reference.

## Material and methods

### Preparation of the reference serum

The VCA-IgA original reference sample was prepared using serum from three highly VCA-IgA positive NPC patients whose diagnosis was histologically confirmed by biopsy. Twenty milliliter blood was collected from each NPC patient and the serum was obtained by centrifugation. The serum was also obtained from the blood of 50 healthy donors and mixed to the pool of the NPC patients to dilute it. The potential presence of VCA-IgA in the serum sample of the healthy donors was tested using the Euroimmun IFA assay and all samples resulted as VCA-IgA negative. All serum samples were also negative for the HIV 1 + 2, HbsAg and Hepatitis C Virus (HCV).

CaCl_2_ was added to the serum of patients and healthy donors to a final concentration of 18 mmol/L. Subsequently, the solution was incubated for 2 h at 37 °C and the insoluble material was removedby centrifugation (30 min at 10000 rpm). Finally, the supernatant was filtered using a 0.2 μm pore size nitrocellulose filter.

The VCA-IgA reference standard was obtained by preparing a series of dilutions of the serum from NPC patients and testing them by ELISA assay from Euroimmun AG (Lubeck,Germany) and CLIA from New Industries (Shenzhen, China). The concentration closer to the upper limit of detection was considered as the best dilution, and corresponding to a target value of 16,000 U/ml.

The bulk reference standard material was obtained by diluting the VCA-IgA–positive NPC serum with the pooled serum from the blood of healthy donors, using the optimal dilution factor as determined above. Finally, 1 liter VCA-IgA bulk reference serum was obtained. Subsequently, the prepared reference serum was divided into 0.2 ml aliquots and stored at-80 °C.

### Patients

The diagnosis of the twenty NPC patients was confirmed by biopsy and consequent histological assay and further tests, including head and neck MRI and chest X-rays. Negativity, low, and medium/high positivity was defined as the result of VCA-IgA titer detected by VCA-IgA Euroimmun IFA. Seven samples were negative (titer< 1:20), seven were low positive (1:20 ≤ titer≤1:80), and six were medium/high positive (titer> 1:80) for VCA-IgA.

During the initial visit all patients were informed that the samples remaining after the routine test might be used for medical research, without involving additional costs and suffering and their written informed consent was obtained. In addition, the ethics committees of the SYSUCC approved this procedure.

### VCA**-**IgA measurement

Six commercial kits were used to analyze the reference and sera from 20 patients with NPC to evaluate the VCA-IgA level. Four ELISA commercial kits produced by the following manufacturers were used: Euroimmun AG (Lubeck, Germany; http://www.oumeng.com.cn/), TarcineBioMed (Beijing, China; http://www.tarcine.com.cn/), Antubio (Zhengzhou, China; http://www.autobio.com.cn/), Beier (Beijing, China; http://www.beierbio.com/). Moreover, 2 CLIA kits produced by the following manufacturers and tested by matching detection systems were used: New Industries Biomedical Engineering (Maglumi 4000 Plus System, Shenzhen, China; http://www.snibe.com/) and YHLO Biotech (iFlash 3000 CLIA System,Shenzhen, China; http://www.szyhlo.com/). Antigen peptides, substrate, incubation time, volume and dilution of the serum, type of conjugate, enzymatic substrate and cut-off level of the six kits were not exactly the same and are listed in Table 1.

**Table 1:** Characteristics of the 7 commercial kits for detecting VCA-IgA antibodies

Manufacturer	Method	Antigen	Chromogenic / luminescence reaction system	Sample dilution	Testing laboratory
Euroimmun	IFA	P3HR1	FITC	1:10	SYSUCC
	ELISA	gp125	HRP/TMB	1:100	SAHGUCM
Beier	ELISA	p18	HRP/TMB	1:10	CHSUMC
Tarcine	ELISA	p18	HRP/TMB	1:10	PHJ
Antu	ELISA	gp125,p18	HRP/TMB	1:10	ATHZU
New Industries	CLIA	p18,p23	ABEI	1:10	SYSUCC
YHLO	CLIA	p18,p23	Acridinium ester/ H2O2	1:10	LMCHCH

*TMB* Tetramethylbenzidine, *ABEI* N-(4-Aminobutyl)-N-ethylisoluminol, *SYSUCC* Sun Yat-sen University Cancer Center, *SAHGUCM* Second Affiliated Hospital of Guangzhou University of Chinese Medicine, *CHSUMC* The cancer hospital of shantou University Medical college, *PHJ* People’s Hospital of Jieyang, *ATHZU* Affiliated Tumor Hospital of Zhengzhou University, *LMCHCH* Liuzhou Maternity and Child Health Care Hospital

The VCA-IgA titer of 20 patients was also tested by IFA considered as the gold standard method.

### Linearity, precision and comparability

The reference serum was serially pre-diluted from 1:1 to 1:64 to measure the sample using the six kits. The linearity of the measurements was calculated by the mean of the linear regression statistical analysis (slope value with 95% CI, and adjusted R^2^) using the original reference, and seven pre-dilutions of the reference serum were obtained and further diluted according to the manufacturer’s protocol of each of the 6 commercial kits (Table 1).

The precision of the kits was evaluated according to the EP15-A2 protocol of the Clinical and Laboratory Standards Institute (CLSI) [17]. The original reference and the 1:16 pre-dilution were analyzed three times per day during a period of five consecutive working days(*n* = 15 per level). The precision of each kit was evaluated as the coefficient of variation (CV), which was calculated from the mean and standard deviation of the data series.

To evaluate the efficacy of harmonization of the antibody levels,the sera from 20 NPC patients with different VCA-IgA concentrations (13 positive and 7 negative) as measured by the IFA were analyzed using 6 commercial kits. The VCA-IgA concentration was calculated by the calibration curve obtained by the original and seven dilutions of the reference serum. A concentration of 16,000 units (U/ml) was arbitrarily assigned to the original reference serum.

### Statistical analysis

Statistical analysis was performed using IBM SPSS Statistics version 19.0 (IBM Corp.). Scatter diagram and linear regression analysis were performed by MedCalc version 11.4.2.0. The VCA-IgA concentration of each of the 20 patients detected by the 6 kits divided into three categories (negative, low and medium/high positive) was compared via randomized block design ANOVA. Then, LSD test was used to perform multiple comparisons between the groups. Quantitative results were correlated by the non-parametric Spearman test. A value of *p* < 0.05 was considered statistically significant.

## Results

### Linearity

**Fig. 1:**
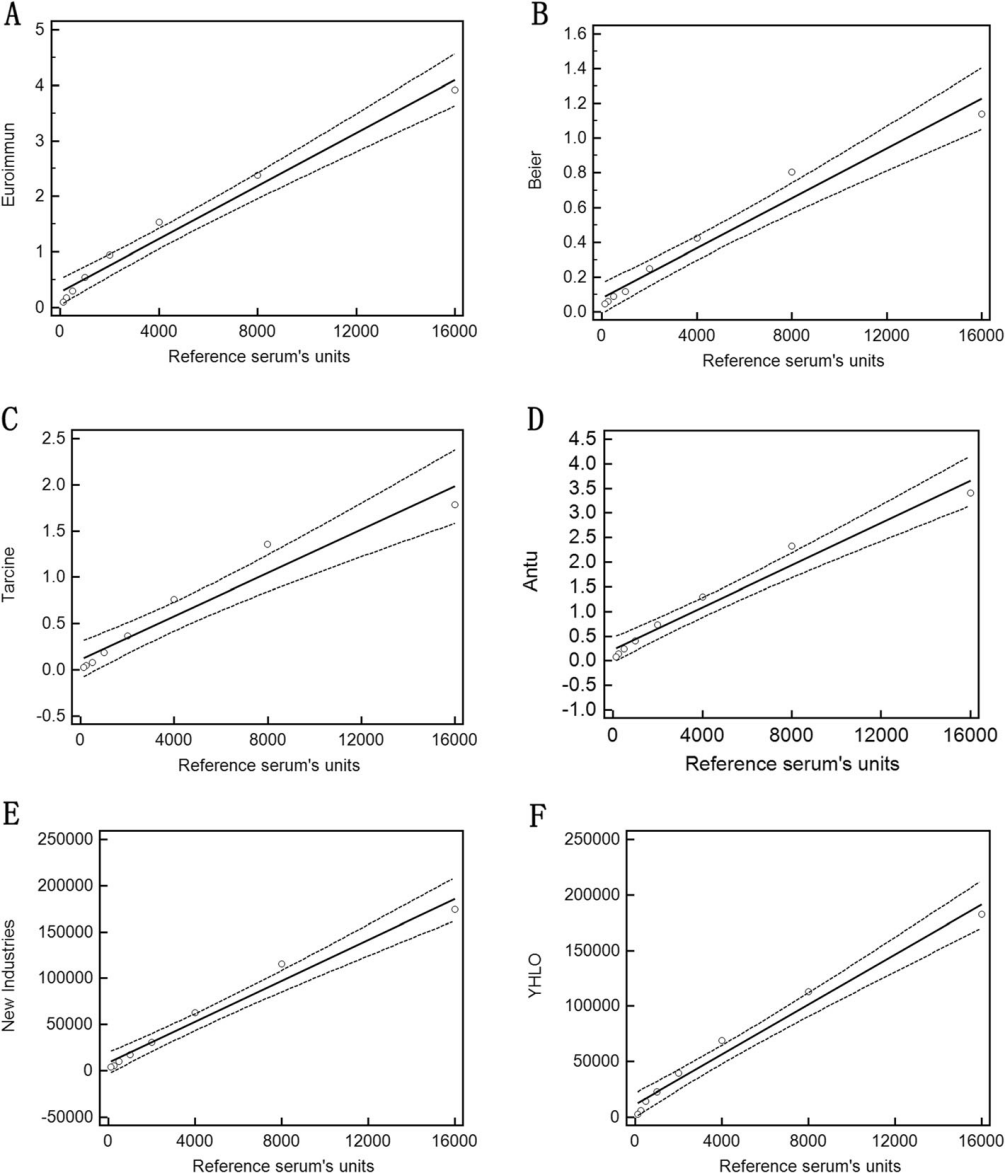
The regression curves obtained with each of the 6 commercial VCA-IgA methods. Values of the ordinate are given in the units used by the assay kit, while the abscissa shows the arbitrary units of the reference sample

The linear regression analysis was performed to evaluate the linearity of the quantitative data. The graphs and correspondent statistical values (slope with 95% CI and adjusted R^2^) are shown in Fig. 1 and Table S1. The coefficient of regression as a measure of linearity of the diluted reference serum tested with each of the 6 commercial kits ranged from 0.00007181 to 11.276 (Table S1). Except for the Tarcine BioMed kit (R^2^ > 0.9367), the regression analysis for the other 5 commercial kits achieved a good correlation coefficient (R^2^ > 0.96) over the entire tested range (Fig. 1,Table S1).

To verify whether the linearity was present even at low and high antibody concentration, one serum sample with a VCA-IgA concentration and another one with a high VCA-IgA concentration were diluted in the same way in which the reference sample was diluted (pre-dilutions from the original concentration to 1:64 followed by the dilution requested in each kit. The results showed that the linearity was maintained even when the antibody concentration was low, but it was not maintained when the antibody concentration was high (Fig. S1).

### Precision

The VCA-IgA concentration detected by Euroimmun and Tarcine kits was calculated using one calibration provided by each kit. The VCA-IgA concentration of New Industries and YHLO kits was calculated by the calibration curve provided by each kit. Since the Antu and Beier ELISA kits did not have a self-made calibrator, the precision was calculated by the OD value of each detection when all the negative and positive controls met the manufacturer requirements. In addition to Beier ELISA kit, other 5 kits resulted in an excellent within-run CVs (CV < 8%) at both low and high VCA-IgA level. Except for Antu and Beier ELISA kits, other 4 kits also resulted in a good total CVs (CV < 10%) (Table 2).

**Table 2:** Levels of VCA-IgA antibodies detected in the VCA-IgA antibody reference serum by the 6 commercial kits

Manufacturer		Cut-off value	Reference serum	Ratio†	Within-run CV		Total CV	
		(arbitrary units)*	(arbitrary units)		High	Low	High	Low
Euroimmun	ELISA	1.1	13.0	11.8	3.98%	6.11%	6.24%	6.52%
Beier**	ELISA	1.1	3.2	2.9	5.03%	19.66%	13.77%	43.11%
Tarcine	ELISA	0.15	1.4	9.3	4.95%	7.58%	8.27%	7.22%
Antu**	ELISA	0.15	3.5	23.3	4.62%	6.45%	13.74%	16.85%
New Industries	CLIA	4	12.0	3.0	2.47%	4.34%	5.95%	9.86%
YHLO	CLIA	1.1	5.6	5.1	4.76%	5.22%	3.98%	4.87%

*VCA-IgA antibody levels detected in the reference serum at the highest concentration tested in each kit

†Ratio was calculated as reference serum arbitrary units divided by manufacturer cutoff value for each kit

**Precision was calculated by OD value of each test when all the negative and positive controls met manufacturer requirements

### Comparability

Table 2 shows the highest VCA-IgA concentrations in the reference serum obtained using the 6 commercial kits, the cut-off values proposed by each manufacturer, and their ratio. The VCA-IgA concentrations varied from 1.4 arbitrary units of the Beier kit to 13 arbitrary units of the Euroimmun kit, thus resulting in a very wide dispersion of the quantitative data.

**Fig. 2:**
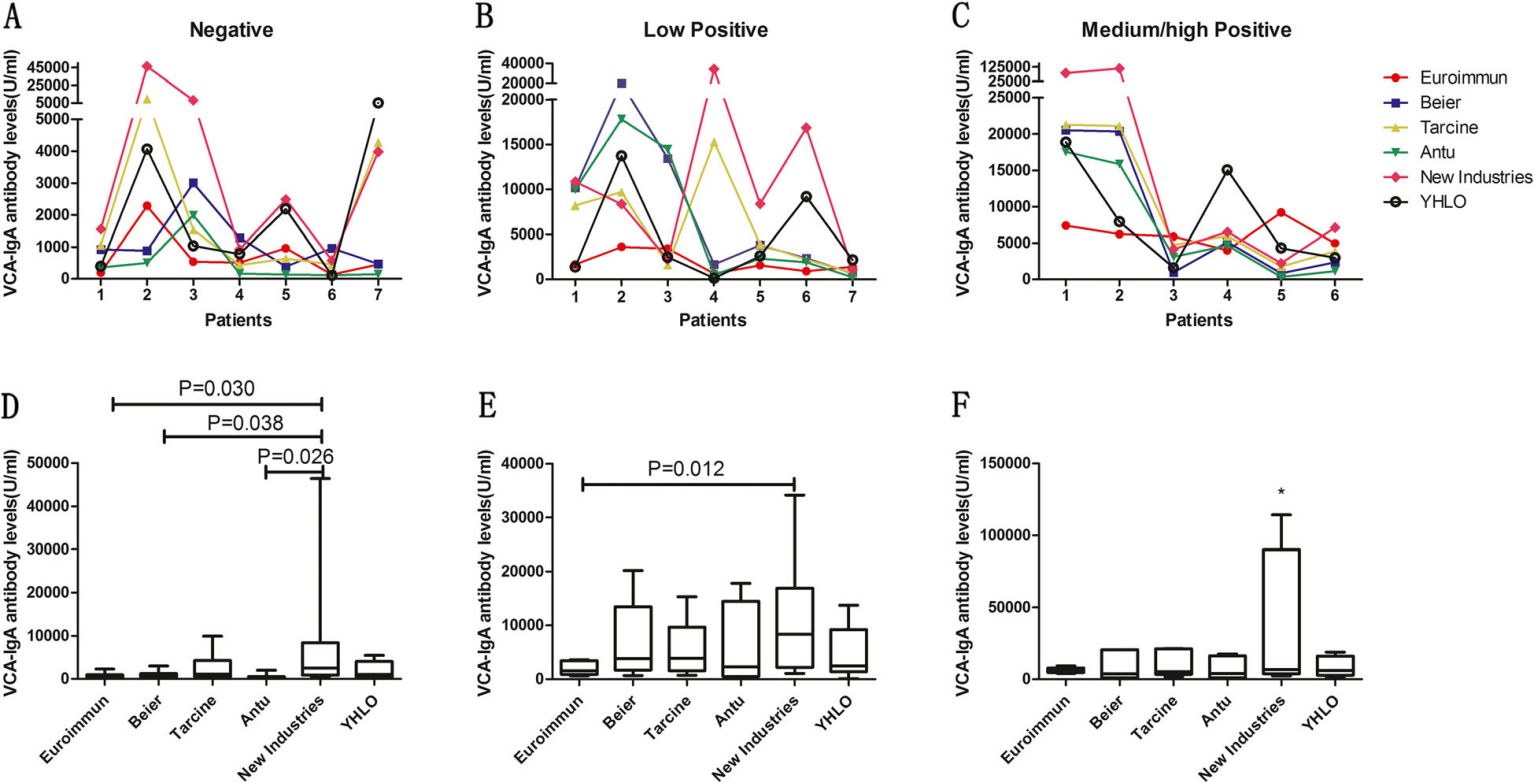
VCA-IgA results of 20 patients calibrated by the reference according three sample categories detected by 6 kits. Line chart of VCA-IgA results detected by each of 6 kits in three sample categories **a** negative; **b** low positive; **c**, medium/high positive. The mean VCA-IgA concentration of each patient by 6 kits divided into three sample categories were used LSD test to make multiple comparisons between the groups. **d**, negative; **e** low positive; **f**, medium/high positive. It has been marked out, when the difference between the two groups is significant (*p* < 0.05). * New Industries group was different from other kits group in medium/high positive categories by LSD test (*p* < 0.05)

The VCA-IgA concentration in each patient obtained using different detecting kits is shown in a line chart (Fig. 2a,b,c). Within the three categories (negative,low and medium/high positive) detected by the 6 kits when the results were obtained by the use of the reference standard, the concentration of VCA-IgA was significantly different among the six kits according to the LSD test(*p* < 0.05) (Fig. 2d,e,f). The value of New Industries and other kits were slightly different. New Industries gave a result different from the one from other kits in the medium/high positive categories by LSD test(*p* < 0.05) (Fig. 2f). This last one was also different from Euroimmun, Beier and Antu kits in the negative category (Fig. 2d), and it was different from the Euroimmun kit in the low positive category (Fig. 2e).

### Correlation among the six assays

The positive VCA-IgA rates in the 20 NPC patients obtained using the six kits were analyzed. The positive rates of each kits were analyzed in 20 NPC patients (Table 3). Euroimmun ELISA resulted inthe highest positive rate in seven kits. The positive and negative rates obtained by the six kits and the IFA considered as the gold standard method are the same, but the differences among the six kits are remarkable (ranging 55–90%) (Table 3).Furthermore,the positive and negative coincidence rates of the four ELISA kits (Euroimmun and Beier and Tarcine and Antubio) were more than 70%. In addition, Beier and New Industries resulted in the highest positive-negative coincidence rate, reaching 90%.

**Table 3:** Performance and coincidence rate of the different assays

Assay	Positive rate(%)^**a**^	Coincidence rate of positive and negative(%)^**b**^					
		Beier	Tarcine	Antubio	New Industries	YHLO	IFA
**Euroimmun**	90	70	75	70	85	75	75
**Beier**	60		75	80	65	55	75
**Tarcine**	65			85	90	60	75
**Antubio**	70				75	55	75
**New Industries**	70					70	75
**YHLO**	70						75
**IFA**	65						

^a^ Positive rate of assay in 20 patients

^b^ Coincidence rate of positive and negative in 20 patients

**Fig. 3:**
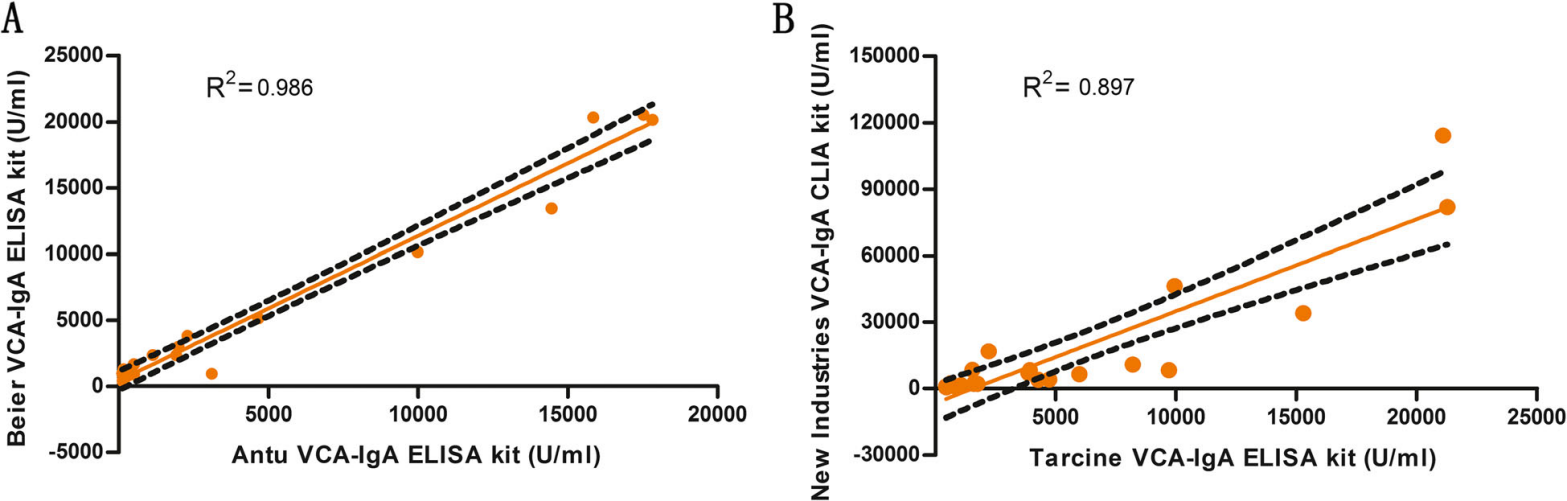
The correlations of VCA-IgA concentrations between different kits. **a** Antu and Beier ELISA kits. **b** Tarcine ELISA kit and New Industries CLIA kit. The low correlation (R^2^ < 0.8) between other kits was not shown

Table 4 shows the Spearman correlation coefficients between the different kits. Almost all of them gave a correlation coefficient lower than 0.80. Although the quantitative results among the different kits are not exactly the same, a good correlation was observed between Antu and Beier ELISA kit (**0.986**), and between Tarcine ELISA kit and New Industries CLIA kit (**0.897**) (Fig. 3).

**Table 4:** Spearman correlations coefficients in the six assays

Manufacturer	Euroimmun	Beier	Tarcine	Antubio	New Industries	YHLO
**Euroimmun**	1	0.494	0.465	0.497	0.440	0.535
**Beier**		1	0.695	**0.986**	0.623	0.641
**Tarcine**			1	0.635	**0.897**	0.546
**Antu**				1	0.542	0.617
**New Industries**					1	0.447
**YHLO**						1

Bold represent results > 0.9

## Discussion

IFA, ELISA and CLIA are three methods for the detection of the VCA-IgA antibody used in clinical laboratories and now available. Numerous manufacturers use different peptides as VCA antigens and different assays, all greatly contributing to the NPC screening. However, the comparison of the results obtained using different kits is necessary to evaluate the best performance and the standardization of the assay is a priority. To this end, a frozen reference sample was prepared and proposed as a candidate serum reference standard forVCA-IgA detection to the Southern China Tumor Markers Standardization Alliance. The main objective of this study was to evaluate the candidate serum reference standard and compare the performance of different commercial ELISA kits and CLIA for the quantitative detection of VCA-IgA.

Our results showed that the reference serum gave positive results in all the commercial VCA-IgA kits used. The linear analysis by the reference standard curve obtained in all the 6 kits showed that the reference sample could be used as a calibrator in different assays using different antigenic substrates. Residual differences may be due to different assay reagents/procedure (including dilution buffer, antigenic peptides, and sample dilution). Since the linearity of the method could not be maintained at a higher VCA-IgA concentration and the linearity(R^2^) did not reach 0.99, it is recommended the use of multi-point curves for each kit in the future.

The precision of each kit is quite different due to the lack of a reference. At present, the VCA-IgA kits can be classified into three categories according to the kit calibrator. The first category that includes Beier and Antu VCA-IgA ELISA kits does not have any calibration material, but only negative and positive quality controls. The results are acceptable when the negative and positive controls are acceptable within a certain range. Therefore, the results within the batch (Within-run CV) may not be very different, but the results of each batch (Total CV) are quite different. No comparison is made among the quantitative results of different test batches. The second category that includes Euroimmun and Tarcine VCA-IgA ELISA kits usesa critical value calibrator with its own. They have satisfactory within-run CV and total CV, but the disadvantage of a single point calibrator is that rODs (OD/cut-off OD) is difficult to accurately express all values because of the imperfect linearity (R2 = 0.9779 and R2 = 0.9367). The third category that includes New Industries and YHLO VCA-IgA CLIA kits used they own calibration curves and provide quantitative results using their own units. Thus, up to now, no systematic evaluation is available on the quantitative and standardized research of all the VCA-IgA methods. Previous studies on VCA-IgA detection generally used ELISA and IFA, [4] CLIA is only related to VCA IgG or IgM antibody. This study is the first including CLIA in VCA-IgA research [18, 19]. Our first study shows that CLIA have better Within-run CV and Total CV. This may be due to the advantages of methodology and matching testing system.

A good agreement with IFA results was obtained using both ELISA and CLIA. The concordance among the 6 kits and IFA for primary NPC diagnosis was 75% overall agreement (Table 3). This result was similar to the one obtained by Karray, who observed 81–91% overall agreement between ELISA (IgA anti-VCA-p18) and IFA for primary NPC diagnosis. They also found a declining reactivity in patients in remission and increasing reactivity in patients with persistent disease or relapse during the follow-up by monitoring the presence of the antibody by both ELISA and IFA [15]. Our previous study also found an excellent correlation and a high degree of concordance between IgA titers (IEA) and rOD levels (ELISA) [2]. Many studies also confirmed that changes in VCA-IgA antibody level can be used for NPC prognosis and screening [13, 20–22]. Pretreatment serum EBV-VCA/IgA titer may be used as an independent prognostic marker of NPC [11]. Liuet al. found that the geometric mean titer of anti-EBV/VCA IgA antibodies before and after radiotherapy was significantly different [23]. People with ascending VCA-IgA antibody titers tend to have higher risks and shorter time to develop NPC, compared to those with a descending pattern [13]. However, Yao et al. did not confirm the role of VCA-IgA as a prognostic biomarker among patients with NPC and undetectable EBV DNA [24]. A possible explanation for these contradictory results may be that the previous study obtained the positive results by IFA, although Yao et al. uses ELISA (Beier kit). In the absence of calibrators, inter-assay differences among ELISA kits may affect the outcome, as explained in the limitations of this article [24] and was confirmed in our study (Total CV =43.11%), confirming once more the importance of the reference in VCA-IgA ELISA detection.

Using the same reference allows connecting different methods or kits to analyze the results that were not previously comparable. This work showed that the same patient had similar results with some different kits, or that the values were different but the trends were the same with different kits (Fig. 2). The results in the 20 patients obtained by the use of the kits calibrated on the reference serum divided into three sample categories showed acertain significant difference in the 6 kits compared via randomized block design ANOVA and further analysis according to LSD test (Fig. 2). The New Industries kit was different from the other kits in medium/high positive category by LSD test(*p* < 0.05). Moreover, New Industries and Euroimmun gave different results in the three groups. The coincidence rate of positive and negative between the six kits were also quite different. (Table 3) This might be due to the different sources of antigens. This aspectis also supported by Gu [25]. The EBV VCA antigen consists of several proteins, such as gp125, p18, and p23. As regards the measurement of IgA, except Euroimmun that uses gp125, other assays use the p18 peptide or other protein mixtures (Antu,New Industries,YHLO). The results depend on the use of a range of different antigens producing different serological responses. Thus, the low correlation between the results obtained using Euroimmunand the other commercial ELISA kit for IgA-VCA might be due to the different antigens. Apart of the use of the same antigen, other factors such as the different dilutions used for the analysis maybe a source of differences [26]. These differences may also lead to a different outcome in the therapeutic and prognostic studies using different VCA-IgA kits. Therefore, in the future, the standards should be divided and unified calibrators should be developed according to the different peptide segments of the antigen protein to achieve anaccurate consistency of the results.

Although shortcomings were still present in the quantitative results among different kits, the improvement of the results obtained in this study was evident. The reference used could reduce the difference among different batches with the same kit. Furthermore, the quantitative results by reference standard curves were more accurate than the semi-quantitative results of IFA in monitoring efficacy and risk. IFA/IEA as the “gold standard” for the detection of IgA to VCA, are laborious techniques, since they are not sufficiently automated to achieve a good level of output, and a certain degree of subjectivity is still present in interpreting the results. The ELISA allows the quantitative detection through the calibration curve, is easy to performand detect quickly a large number of samples. Finally, the results of different manufacturers’ kits were compared by the use of a reference standard. A good correlation (**correlations coefficient = 0.986**) was obtained between Antu and Beier ELISA kits in the results obtained by standard curves using the reference standard.

## Conclusion

In conclusion, the use of the serum as the reference might reduce the differences among different kits and allow a comparison among different kits. Our subsequent studies should focus on the diversity between different antigens and the attempt to unify them to promote the further development of VCA-related research.

## Supplementary information

**Additional file 1: Table S1.** A summary of linear regression studies **Figure S1.** The regression curves obtained by diluted one more low and high VCA-IgA serum concentrations with each of the 6 commercial methods. Values of the ordinate are givenin the units used by the assay kit, while the abscissa shows the arbitrary units of the reference sample.

## Data Availability

The datasets used and/or analysed during the current study are available from the corresponding author on reasonable request.

## Abbreviations

NPC: Nasopharyngeal carcinoma
VCA: Virus capsid antigen
IgA: Immunoglobulin A
CLIA: Chemiluminescent immunoassays
EBNA1-IgA: Epstein-Barr virus nuclear antigen 1
IFA: Indirect immunofluorescence
ELISA: Enzyme-linked immunosorbent assay
EBV: Epstein-Barr virus
EA: Early antigen
IEA: Immunoenzymatic assay
WHO: World Health Organization
ASIRW: Age-standardized incidence rates of world
SYSUCC: Sun Yat-sen University Cancer Center

## References

[1] Chen W, Sun K, Zheng R, Zeng H, Zhang S, Xia C (2018). Cancer incidence and mortality in China, 2014. Chinese J Cancer Res= Chung-kuo yen cheng yen chiu.

[2] Chen H, Chi P, Wang W, Li L, Luo Y, Fu J (2014). Evaluation of a semi-quantitative ELISA for IgA antibody against Epstein-Barr virus capsid antigen in the serological diagnosis of nasopharyngeal carcinoma. Int J Infect Dis.

[3] Chen H, Chen S, Lu J, Wang X, Li J, Li L (2017). Multiparametric detection of antibodies against different EBV antigens to predict risk for nasopharyngeal carcinoma in a high-risk population of China. Cancer Prev Res (Phila).

[4] Liu Y, Huang Q, Liu W, Liu Q, Jia W, Chang E (2012). Establishment of VCA and EBNA1 IgA-based combination by enzyme-linked immunosorbent assay as preferred screening method for nasopharyngeal carcinoma: a two-stage design with a preliminary performance study and a mass screening in southern China. Int J Cancer.

[5] Henle G, Henle W (1976). Epstein-Barr virus-specific IgA serum antibodies as an outstanding feature of nasopharyngeal carcinoma. Int J Cancer.

[6] Chien YC, Chen JY, Liu MY, Yang HI, Hsu MM, Chen CJ (2001). Serologic markers of Epstein-Barr virus infection and nasopharyngeal carcinoma in Taiwanese men. N Engl J Med.

[7] Zeng Y, Zhang LG, Li HY, Jan MG, Zhang Q, Wu YC (1982). Serological mass survey for early detection of nasopharyngeal carcinoma in Wuzhou City, China. Int J Cancer.

[8] Zong YS, Sham JS, Ng MH, Ou XT, Guo YQ, Zheng SA (1992). Immunoglobulin a against viral capsid antigen of Epstein-Barr virus and indirect mirror examination of the nasopharynx in the detection of asymptomatic nasopharyngeal carcinoma. Cancer.

[9] Chen Y, Xin X, Cui Z, Zheng Y, Guo J, Lin Y (2016). Diagnostic value of serum Epstein-Barr virus capsid antigen-IgA for nasopharyngeal carcinoma: a meta-analysis based on 21 studies. Clin Lab.

[10] Li S, Deng Y, Li X, Chen QP, Liao XC, Qin X (2010). Diagnostic value of Epstein-Barr virus capsid antigen-IgA in nasopharyngeal carcinoma: a meta-analysis. Chin Med J (Engl).

[11] Ling W, Cao SM, Huang QH, Li YH, Deng MQ (2009). Prognostic implication of pretreatment titer of serum immunoglobulin a against Epstein-Barr virus capsid antigen in nasopharyngeal carcinoma patients in Sihui, Guangdong. Ai zheng = Aizheng = Chinese journal of cancer.

[12] Zeng Y (1985). Seroepidemiological studies on nasopharyngeal carcinoma in China. Adv Cancer Res.

[13] Cao SM, Liu Z, Jia WH, Huang QH, Liu Q, Guo X (2011). Fluctuations of epstein-barr virus serological antibodies and risk for nasopharyngeal carcinoma: a prospective screening study with a 20-year follow-up. PLoS One.

[14] Du JL, Chen SH, Huang QH, Xie SH, Ye YF, Gao R (2016). Subtype distribution and long-term titer fluctuation patterns of serum anti-Epstein-Barr virus antibodies in a non-nasopharyngeal carcinoma population from an endemic area in South China: a cohort study. Chin J Cancer.

[15] Karray H, Ayadi W, Fki L, Hammami A, Daoud J, Drira MM (2005). Comparison of three different serological techniques for primary diagnosis and monitoring of nasopharyngeal carcinoma in two age groups from Tunisia. J Med Virol.

[16] Gao R, Wang L, Liu Q, Zhang LF, Ye YF, Xie SH (2017). Evaluation of seven recombinant VCA-IgA ELISA kits for the diagnosis of nasopharyngeal carcinoma in China: a case-control trial. BMJ Open.

[17] Wu X, Chao Y, Wan Z, Wang Y, Ma Y, Ke P (2016). A comparative evaluation of the analytical performances of Capillarys 2 flex piercing, Tosoh HLC-723 G8, premier Hb9210, and Roche Cobas c501 Tina-quant gen 2 analyzers for HbA1c determination. Biochem Med.

[18] Grandjean Lapierre S, Vallieres E, Rabaamad L, Labrecque M, Chartrand C, Renaud C (2016). Evaluation of the abbot architect() epstein-barr virus viral capsid antigen IgM, viral capsid antigen IgG and nuclear antigen IgG assays in a pediatric and adult population. J Clin Virol.

[19] Francois C, Segard C, Bouvier M, Stefanski M, Pannier C, Zawadzki P (2018). Comparison of Abbott architect((R)), Siemens Immulite((R)), and Diasorin liaison((R)) for determination of Epstein-Barr virus serological diagnosis. Diagn Microbiol Infect Dis.

[20] de Vathaire F, Sancho Garnier H, de The H, Pieddeloup C, Schwaab G, Ho JH (1988). Prognostic value of EBV markers in the clinical management of nasopharyngeal carcinoma (NPC): a multicenter follow-up study. Int J Cancer.

[21] Lynn TC, Tu SM, Kawamura A (1985). Long-term follow-up of IgG and IgA antibodies against viral capsid antigens of Epstein-Barr virus in nasopharyngeal carcinoma. J Laryngol Otol.

[22] Ji MF, Wang DK, Yu YL, Guo YQ, Liang JS, Cheng WM (2007). Sustained elevation of Epstein-Barr virus antibody levels preceding clinical onset of nasopharyngeal carcinoma. Br J Cancer.

[23] Liu MT, Yeh CY (1998). Prognostic value of anti-Epstein-Barr virus antibodies in nasopharyngeal carcinoma (NPC). Radiat Med.

[24] Yao JJ, Lin L, Jin YN, Wang SY, Zhang WJ, Zhang F (2017). Prognostic value of serum Epstein-Barr virus antibodies in patients with nasopharyngeal carcinoma and undetectable pretreatment Epstein-Barr virus DNA. Cancer Sci.

[25] Gu AD, Mo HY, Xie YB, Peng RJ, Bei JX, Peng J (2008). Evaluation of a multianalyte profiling assay and an enzyme-linked immunosorbent assay for serological examination of Epstein-Barr virus-specific antibody responses in diagnosis of nasopharyngeal carcinoma. Clin Vaccine Immunol.

[26] de Ory F, Guisasola ME, Sanz JC, Garcia-Bermejo I (2011). Evaluation of four commercial systems for the diagnosis of Epstein-Barr virus primary infections. Clin Vaccine Immunol.

