# Supplementary material for: Preliminary evaluation of a candidate international reference for Epstein–Barr virus capsid antigen immunoglobulin A in China
Source: Infect Agent Cancer. 2020 Apr 29;15:25. doi: 10.1186/s13027-020-00294-8 (PMC7191735; doi:10.1186/s13027-020-00294-8)
Supplement: Supplementary file 1 — Additional file 1: Table S1. A summary of linear regression studies Figure S1. The regression curves obtained by diluted one more low and high VCA-IgA serum concentrations with each of the 6 commercial methods. Values of the ordinate are givenin the units used by the assay kit, while the abscissa shows the arbitrary units of the reference sample. [file 13027_2020_294_MOESM1_ESM.pdf]

Table S1. A summary of linear regression studies

Manufacturer	Slope(95% CI)	Coefficient of determination R^2	Residual standard deviation
Euroimmun	2.39E-04 (2.03E-04 ~ 2.74E-04)	0.978	0.214
Beier	7.18E-05 (5.83E-05 ~ 8.54E-05)	0.966	0.081
Tarcine	1.17E-04 (8.67E-05 ~ 1.47E-04)	0.937	0.182
Antu	2.15E-04 (1.76E-04 ~ 2.53E-04)	0.968	0.232
New Industries	11.0757 (9.2955 ~ 12.8558)	0.975	10650.775
YHLO	11.276 (9.6405 ~ 12.9114)	0.979	9784.835

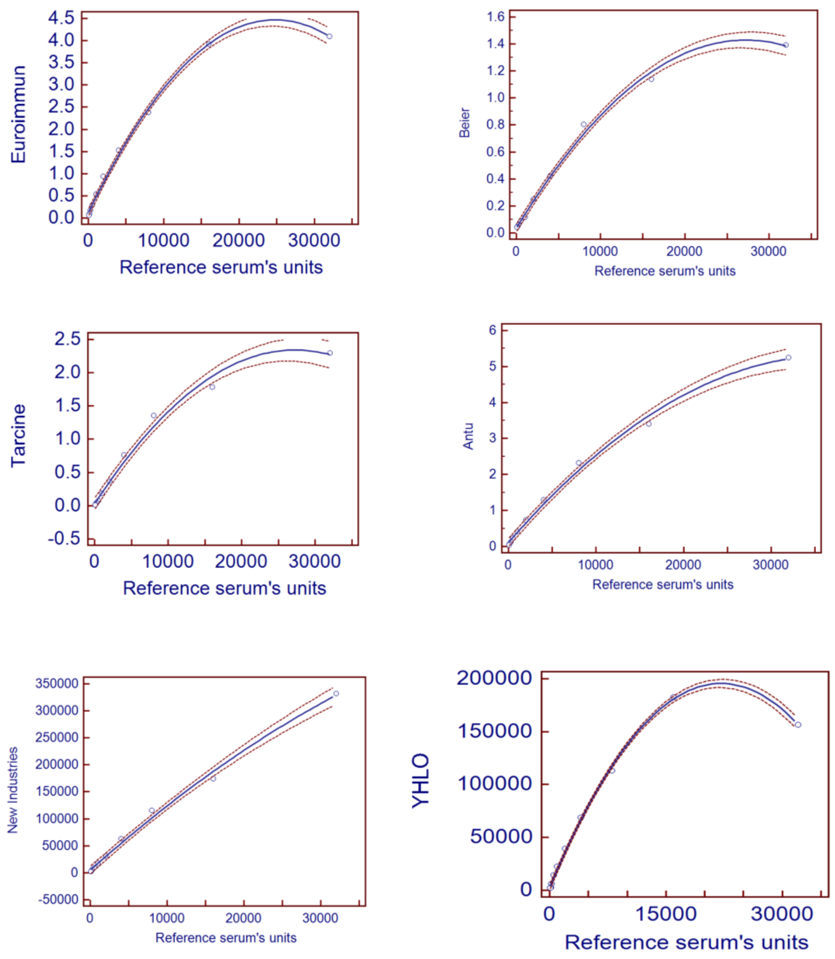

Figure S1. The regression curves obtained by diluted one more low and high VCA-IgA serum concentrations with each of the 6 commercial methods. Values of the ordinate are given in the units used by the assay kit, while the abscissa shows the arbitrary units of the reference sample.
